# Spinning Through History: Evolution of the Concept of Vestibular Migraine

**DOI:** 10.1097/ONO.0000000000000040

**Published:** 2023-09-12

**Authors:** Yoon-Hee Cha

**Affiliations:** 1 Department of Neurology, Minneapolis, MN

**Keywords:** Meniere’s disease, migraine, vertigo, vestibular migraine

## Abstract

Vestibular migraine represents a growing public health problem, imposing enormous societal burdens in the form of patient suffering, loss of productivity, and direct healthcare costs. This raises the question of how we developed our ideas about vestibular migraine and how these ideas shape how we treat it.

This review walks through the history of how our conceptualization of migraine and vestibular symptoms evolved, starting with clinical observations in ancient times, inclusion under the umbrella of Meniere’s disease, and then separation from Meniere’s disease with its own identity. Tradition, clinical observations, and diagnostic criteria developed by professional societies have played prominent roles in building our current concept of vestibular migraine. A review of the ideas that have shaped our current conception of vestibular migraine may help us to see which ones have stood the test of time and which ones should continue to evolve. As in other disciplines, we study history in medicine to be inspired, warned, and sometimes, to be freed.

**Highlights**

1. Cerebral congestion was the predominant mechanism evoked for paroxysmal neurological symptoms, including vertigo, until the mid-19th century.
2. In 1861, Prosper Meniere showed evidence for inner ear involvement in the generation of vertigo, defying the cerebral congestion theory.
3. Episodic vertigo was originally conceptualized under the Meniere’s umbrella in the early 20th century then moved to under the migraine umbrella in the early 21st century as vestibular migraine.
4. The current diagnostic criteria for vestibular migraine were developed as a collaboration between the International Headache Society and the International Bárány Society.

Vestibular migraine has quickly become the most common diagnosis in specialty practices caring for patients with headaches and vestibular symptoms, now accounting for 25% of patients in headache clinics and up to 30% of patients in neurotology clinics (1–4). What this high prevalence of vestibular migraine reflects is how common dizziness and vestibular symptoms are in people who are prone to headaches and how much the right framework for understanding their association is needed.

The current diagnostic criteria for definite and probable vestibular migraine jointly, that was created by the International Classification of Headache Disorders (ICHD) and the International Bárány Society, were built off the criteria for migraine headaches, a primary headache disorder (5,6) (Table 1). The presumption is that vestibular migraine is itself a primary disorder without a structural cause. Yet, vestibular migraine symptoms heavily overlap with those of Meniere’s disease, a disorder of known structural inner ear dysfunction. Ambiguity in which category of disorders vestibular symptoms with headache should be placed has led to diagnostic and management unease, especially when there are subjective symptoms such as tinnitus but no objective abnormalities such as hearing loss.

**TABLE 1.** ICHD-3 and Barany Society Criteria for Vestibular Migraine

1. Definite vestibular migraine
A. At least 5 episodes with vestibular symptoms of moderate or severe intensity, lasting 5 min to 72 hrs
B. Current or previous history of migraine with or without aura according to the International Classification of Headache Disorders (ICHD)
C. One or more migraine features with at least 50% of the vestibular episodes:
Headache with at least 2 of the following characteristics: one-sided location, pulsating quality, moderate or severe pain intensity, aggravation by routine physical activity
Photophobia and phonophobia
Visual aura
D. Not better accounted for by another vestibular or ICHD diagnosis
2. Probable vestibular migraine
A. At least 5 episodes with vestibular symptoms of moderate or severe intensity, lasting 5 min to 72 hours
B. Only 1 of the criteria B and C for vestibular migraine is fulfilled (migraine history or migraine features during the episode)
C. Not better accounted for by another vestibular or ICHD diagnosis

Vestibular symptoms include: spontaneous vertigo of either self or visual surround motion, positional vertigo, visually-induced vertigo, head motion-induced vertigo, and head motion-induced dizziness with nausea.

Adding to the complexity is that vestibular migraine criteria allow for a variety of symptoms, including spontaneous vertigo of either self or visual surround motion, positional vertigo, visually-induced vertigo, head motion-induced vertigo, and head motion-induced dizziness with nausea. Allowable symptoms can last from 5 minutes to 72 hours, representing a wide parameter space (5). Understanding how we developed our current ideas of vestibular migraine may be useful in deciding which paradigms for vestibular symptoms and headaches are the most salient for treatment and perhaps even prevention of new cases. To that end, this review will describe the evolution of our current concept of vestibular migraine. Of note, while the International Classification of Vestibular Disorders developed by the Bárány Society defined dizziness and vertigo to be distinct symptoms (dizziness as spatial disorientation and vertigo as an illusion of motion), for the purpose of this review, they will be used interchangeably as vestibular symptoms have historically been referred to by either word (7).

## PREVALENCE AND BURDEN

Neuhauser et al. (9) in Germany first developed criteria for “migrainous vertigo,” a term that evolved into vestibular migraine. They found that 7% of patients in their dizziness clinics and 9% of patients in their headache clinics met the criteria for migrainous vertigo (8). They estimated the population 1 year and lifetime prevalence of migraine assessed by telephone interview of 4869 people as 0.89% and 0.98%, respectively.

The most recent assessment of vestibular migraine in the United States puts the prevalence number even higher at 2.7%, of which 75.8% are female (10). In this study, Formeister used 2008 census data and retroactively applied the 2012 Bárány Society criteria for vestibular migraine to a sample of 21,871 people who had completed a 210-item Balance and Dizziness Supplement based on 74,236 total respondents (10). The risk factors for vestibular migraine were found to be age <40 years, female sex, anxiety, depression, and prior head trauma. Extrapolation of the prevalence estimates from this study to the 2023 census would yield over 9 million people suffering from vestibular migraine in the United States (11).

Despite the large numbers, the overall burden of vestibular migraine may be underreported. It has only been in recent years that there has been the development of a handicap inventory created specifically for vestibular migraine (VM-PATHI: Vestibular Migraine Patient Assessment Tool and Handicap Inventory) that directly queries the experience of patients with combined headache and vestibular symptoms (12,13). A growing number of studies have shown that the risk of falls and nonvestibular symptoms such as cognitive dysfunction and chronic fatigue are significantly higher in patients with vestibular migraine than in those without (14–16). The complexity of symptoms in vestibular migraine explains the high annual cost of caring for these patients, estimated at $11,371 compared to $9,579 for Meniere’s disease and $8,247 for Benign paroxysmal positional vertigo (17). Considering the chronicity of vestibular migraine and the young age of onset, these values represent a significant national economic and public health burden.

## THE CEREBRAL CONGESTION MODEL OF VERTIGO

The connection between headache and vertigo was made at least as far back as Hippocrates (BCE 470-360). “*… south winds relax the body, make the tissues moist, reduce acuity of hearing, and produce headaches and vertigo*.” Aphorisms Sect 3:17 (18). Hippocrates’, “four humors,” model of human illness (blood, black bile, yellow bile, and phlegm), was continued by Galen of Pergamum (CE 129–200), the chief authority on medicine and biology until the 19th century credited for creating the term “hemicrania” from which the word “migraine,” derives. In his work, *De locis affectis*, (“On the affected parts”), he attributed *kephalalgia* (headache) to the retention of yellow bile in the stomach, the headache being relieved with vomiting. *Skotoma* referred to one of several forms of vertigo that leads to impaired and sometimes darkened vision (19).

Galenic principles were carried into the Middle East and systematized by the Persian scholar Ibn Sina (Latin, Avicenna, CE 980–1037). In the Canon of Medicine, Ibn Sina described vertigo as, “a brain disorder but as principle symptom of melancholy with ‘trembling,’ ’fear,’ and noises in the ear,” and later, “the patient feels ill and uneasy, and suffers from impaired vision, dizziness, and migrainous headaches, with yawning and stretching…” (20).

The prevailing belief for more than 2 millennia after Hippocrates was that vertigo was due to fluid imbalances, specifically “apoplectiform cerebral congestion” caused by the overfilling of blood vessels in the brain. This process was summarized in the “Tractatus de Apoplexia” by Francisco Bayle in 1678 and formed the basis of disease paradigms well into the mid-19th century (21,22). Disorders such as vertigo, strokes, and epilepsy were thought to be due to compressed vessels or blockage by phlegm and were thus treated with bloodletting, leeches, laxatives, and purgatives (19,20).

The cerebral congestion theory formed the foundation of neurological care in the United States. It was the main theme of the first textbook on neurological disorders published in 1871 by William Hammond (CE 1828–1900) the Surgeon General during the Civil War who became the first specialist in neurology in the United States (23–25). In his book, he described “active cerebral congestion,” from arterial compression resulting in vertigo, “In some cases, headache constitutes the chief feature of the disorder and is almost constantly present. There are noises, such as roaring, rumbling, and ringing, in the ears…” Passive cerebral congestion due to venous compression would lead to, “Vertigo, pain, illusions, hallucinations, and delusions, are nevertheless generally present at one time or another. But, the stupor or tendency to somnolence is the most prominent feature…” (23).

## THE INNER EAR INJURY MODEL OF VERTIGO

Two thousand years of assumptions about the origins of vertigo would be uprooted by Prosper Meniere (CE 1799–1862). Meniere was a French physician who trained at the prestigious hospital Hôtel-Dieu with the surgeon Baron Guillaume Dupuytren (CE 1777–1835). He was well-known for his obstetrical surgical skills and for his writing. Despite having no background in otology, he was appointed as the head of the Hospital for Deaf Mutes in 1838 when the holder of that position, Jean Marc Gaspard Itard (CE 1775–1838) died (26,27). In this new role, Meniere observed that deaf patients, already accepted to have inner ear disease, often experienced vertigo.

In January 1861, Meniere presented a paper to the Paris Imperial Academy of Medicine entitled “Sur une forme de surdité grave dépendant d‘une lesion de lòreille interne,” (“On a serious form of deafness dependent on a lesion of the inner ear”) (28,29). He described a patient who had experienced episodes of vertigo, tinnitus, and hearing loss but had no evidence of cerebral disease. This not only questioned the cerebral congestion theory but also the exuberant treatments that were based on this theory. The talk was not well-attended but became part of a lively debate when, the following week, Armand Trousseau (CE 1820–1910), Physician-in-Chief at Hôtel-Dieu, presented a paper questioning the prevailing belief that epilepsy was due to apoplectiform cerebral congestion. Trousseau’s and Meniere’s claims raised skepticism and vehement opposition, resulting in a debate period that was truncated due to fruitlessness and exasperation (27,30).

Undeterred, Meniere published a paper in September 1861 entitled, “Mémoire sur des lésions de l’orielle interne donnant lieu à des symptômes de congestion cérébrale apoplectiforme,’ (A Report on Lesions of the Inner Ear Giving Rise to Symptoms of Cerebral Congestion of Apoplectic Type,”) in which he extended a report that he had previously made on a young patient who had experienced sudden hearing loss and vertigo shortly before death and had been found to have a “bloody exudate,” in the inner ear (31). He concluded the paper with this statement, quite controversial for its time, “All the evidence indicates that the organic lesion which produces these disturbances of function is situated in the semicircular canals” (31).

Meniere died in 1862 from pneumonia before seeing the widespread acceptance of his ideas. By as early as 1872, however, the triad of vertigo, tinnitus, and hearing loss became known as, “Maladie de Meniere” (32),. Although he had not used the word, “hemorrhage,” Meniere’s original description of the “bloody exudate,” led to the assumption that Meniere’s disease was due to inner ear hemorrhage well into the 20th century (33). This idea would be challenged by Kyoshiro Yamakawa in Japan and the team of Charles Hallpike and Hugh Cairns in England, who independently reported in 1938 that endolymphatic hydrops (without hemorrhage) characterized Meniere’s disease (34–36). Work over the following century would introduce additional mechanisms, such as autoimmunity and genetic contributions to Meniere’s pathogenesis (37).

## MIGRAINE CRITERIA AND VESTIBULAR SYMPTOMS

Shortly after the introduction of, “Maladie de Meniere,” Edward Liveing published his seminal work on migraines entitled, “On Megrim: Sick-Headaches,” which included prominent sections on dizziness (38). Among other manifestations, he described visually-induced dizziness, spinning vertigo, orthostatic intolerance, and the phenomenon of vection in migraines.

Vestibular symptoms as a formal feature of migraine were solidified in 1961 when Bickerstaff reported on patients who experienced vertigo in the context of what he termed “basilar artery migraine” (39). The idea of a basilar artery localization of vertigo was carried forward in the 1988 publication of the International Classification of Headache Disorders-1 (ICHD-1), which included vestibular symptoms in the context of, “basilar migraine.” This was softened to “basilar-type migraine,” in ICHD-2 in 2004 as there was a lack of evidence for basilar artery involvement in the pathogenesis of vestibular symptoms in migraine. “Brainstem-type,” symptoms were subsequently categorized under “migraine with brainstem aura,” in ICHD-3 beta in 2013 and in the final form in 2018 (6,40,41). ICHD-3 also introduced the diagnosis of “vestibular migraine,” in Appendix A1.6.6.

There has been an understandable concern that the definition of migraine with brainstem aura is overly broad and overestimates the true prevalence of auras that are actually referable to the brainstem and are not potentially some other disorder (42). With these new criteria, the boundaries between Meniere’s attacks with migraine, migraine with brainstem aura, and vestibular migraine became somewhat blurry since Meniere’s attacks are frequently associated with migraine headaches (43,44).

## MENIERE’S CRITERIA AND VESTIBULAR SYMPTOMS

The criteria for Meniere’s disease similarly underwent an evolution, starting with the American Academy of Ophthalmology and Otolaryngology Committee on Hearing and Equilibrium proposal in 1972 of 3 categories of Meniere’s disease: 1) Classic Meniere’s: vertigo with unilateral tinnitus and hearing loss, 2) Vestibular Meniere’s: vertigo without auditory symptoms, and 3) Cochlear Meniere’s: progressive but fluctuating hearing loss, tinnitus, and aural fullness without vertigo (45).

It was observed, however, that while both vestibular Meniere’s and cochlear Meniere’s could evolve to become the full syndrome of classical Meniere’s, they could remain as isolated syndromes for decades (46,47). Thus, in 1985, the committee dropped the latter 2 categories and kept Classic Meniere’s (48).

In 1995, the classic Meniere’s diagnosis was divided into “Certain,” “Definite,” “Probable,” and “Possible,” subcategories. The diagnosis of “certain,” could only be made by histopathologic correlation, which presented practical problems as it required surgical removal of the inner ear. “Possible Meniere’s,” could be diagnosed based on, “episodic vertigo of the Meniere’s type,” without the hearing loss. This opened the door for many episodic vestibular symptoms not otherwise defined to be placed under Meniere’s umbrella, including syndromes that would later be defined as vestibular migraine (49).

In 2015, the International Bárány Society and the American Academy of Otolaryngology-Head and Neck Surgery modified the criteria for Meniere’s disease to include only the “Definite,” and “Probable,” categories. Both required at least 2 episodes of vertigo, tinnitus, or aural fullness, but only “Definite,” Meniere’s required documented hearing loss (5,50,51). The diagnostic criteria for vestibular migraine had been published in 2012, with the stipulation that it should be diagnosed when “Not better accounted for by another vestibular or ICHD diagnosis” (5). Thus, “Probable Meniere’s,” and vestibular migraine became separated by the presence of hearing loss, aural symptoms, duration of vertigo, and the greater range of vestibular symptoms allowed in vestibular migraine.

Of note, while vestibular dysfunction measured by caloric responses is generally reported to be higher in Meniere’s disease (up to 58%) than vestibular migraine (up to 25%), they overlap in range so much that peripheral vestibular dysfunction cannot be used as a criterion to distinguish between these diagnoses (44,52–54). The overlap is even greater when considering less specific signs of asymmetric vestibular tone, such as head-shaking nystagmus (54). These differences should be viewed in the context of the reported high baseline rate of peripheral vestibular dysfunction in patients with migraine headaches who do not experience vertigo (55).

## THE MIGRAINE−MENIERE’S CONNECTION

The link between migraine headaches and vertigo is so strong that Meniere, who suffered from migraine headaches himself, said this: “I do not hesitate to regard these migraines as dependent upon a lesion of the inner ear; they are accompanied by noises, by vertigo, by gradual diminution of hearing, and most often their deafness resists all methods of treatment” (29,56). We can be reasonably sure that migraine headaches do not *originate* in the inner ear but we do not yet know what common pathology affects both the inner ear and pain pathways in the migraine cascade.

Studies through the years have examined the prevalence of migraine in Meniere’s disease and more specifically in recurrent vertigo without hearing loss. Radtke et al found a higher rate of migraine in Meniere’s patients (56%) than controls (25%) (57). Ghavami et al reported a 51% rate of migraine in Meniere’s patients including 41% that also met the criteria for vestibular migraine (58). Ray et al (59) reported a rate of 45% of migraine in Meniere’s patients versus 9% in other vestibular disorders. Cha et al showed that patients with Meniere’s disease and migraine were more likely to be female, have an earlier age of onset of Meniere’s symptoms, have bilateral hearing loss, and have a positive family history of both migraine headaches and vertigo than patients with Meniere’s disease alone (60). This was confirmed by Clemmens and Ruckenstein, who showed that patients with bilateral Meniere’s disease were more likely to be female, be younger, and have a personal history of migraines than patients with unilateral Meniere’s disease (61).

Over time, the relevance of migraine headaches in otherwise spontaneous attacks of vertigo became clearer. In their 1984 paper entitled, “The Neurotological Manifestations of Migraine,” Kayan and Hood (62) showed that while the general sense of “giddiness,” differed only slightly, episodes of actual vertigo were over 3 times higher and motion sickness over 2.5 times higher in patients with migraine headaches than those with tension headaches. Rassekh and Harker found that patients with classical Meniere’s disease (with hearing loss) had a much lower rate of migraine at 22% than patients with “vestibular Meniere’s,” (vertigo without hearing loss) at 81% (63). Brantberg and Baloh’s study found a 51% rate of migraine in benign recurrent vertigo patients (vertigo without hearing loss) but only 21% in classical Meniere’s patients (43). Cha et al (64) found a migraine prevalence of 87% in patients with benign recurrent vertigo when the latter was defined as at least 2 spells of spontaneous vertigo.

Altogether, though the rate of migraine in Meniere’s has varied owing to differences in criteria for migraine and Meniere’s disease over time, the prevalence of migraine headaches in patients with recurrent vertigo both with and without hearing loss has usually been shown to be much higher than the population baseline rate of migraine, typically reported as about 6%–18% (43,64,65). However, the migraine incidence in Meniere’s has also occasionally been reported to be near baseline population levels and even lower than controls, raising concerns about ascertainment bias and the effect of changing criteria affecting the categorization of overlapping features (63,66).

## EVOLUTION OF TERMINOLOGY

**FIG 1.**
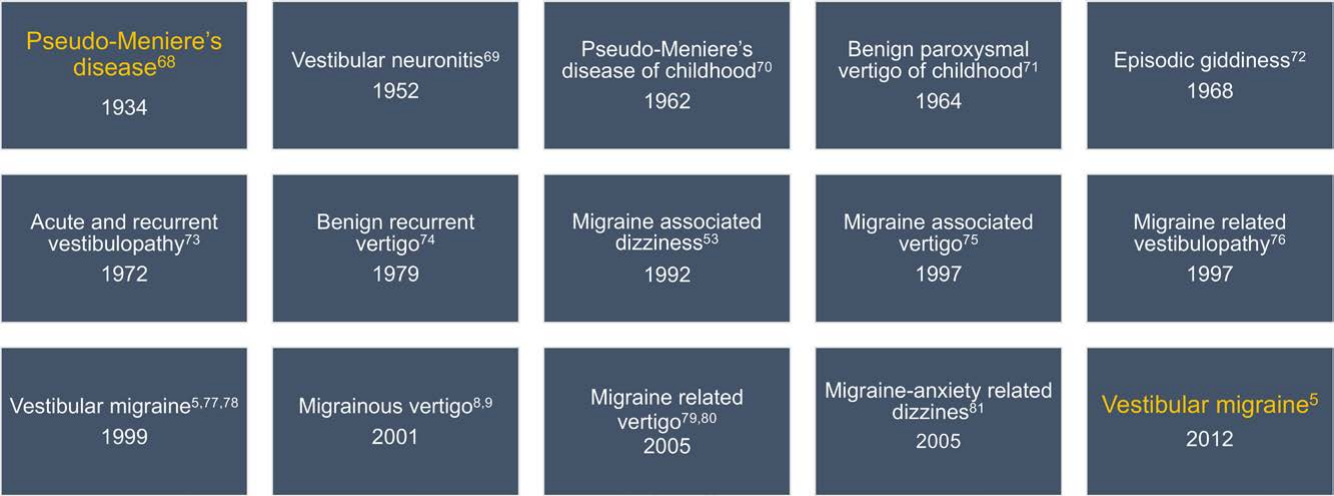
Evolution of terminology for episodic vestibular syndromes between Pseudo-Meniere’s disease and vestibular migraine. Note that terms were used concurrently with a large span of overlap. They are listed according to the earliest reference in the most cited papers.

Descriptions of Meniere’s attacks before they were called by this name frequently mention headaches or head pressure during the triad of vertigo, tinnitus, and hearing loss (67). Efforts to define an episodic vertigo syndrome without hearing loss led to an outpouring of terms that were not the full Meniere’s triad (5,8,53,68–81). The evolution of these terms shows the gradual movement of episodic vertigo spells away from under the umbrella of Meniere’s disease (a structural inner ear disorder) to under the umbrella of migraine (a primary headache disorder) (**Fig. 1**).

## MECHANISM AND TREATMENT

A variety of mechanisms for vestibular migraine have been invoked through the years, such as spreading depression in the brainstem, the release of Calcitonin gene-related peptide (CGRP), vascular processes (vasoconstriction, ischemia, and arteriovenous shunting), and secondary hydrops from damage to the inner ear (53,82–84). Widespread cortical vestibular projections may raise the chances that vestibular symptoms could be caused by a multitude of factors that affect cortical function (85,86). Those factors could be anything from sleep, diet, activity, sunlight, chemicals, visual stimulation, and even cognitive load.

Treatments for vestibular migraine have so far relied on those for migraine headaches with the addition of vestibular rehabilitation. The most common medications used to treat vestibular migraine include calcium channel blockers (verapamil and flunarizine), beta-blockers (metoprolol and propranolol), selective serotonin reuptake inhibitors (venlafaxine), tricyclic amines, and a variety of anticonvulsants (topiramate and lamotrigine) (87). There are suggestions that the more recent anti-CGRP medications can be effective (88,89). So far, there has been no support for any therapy being superior to any other, making the choice of treatment based on factors such as patient tolerance and comorbid conditions (87,88,90–92). Treatments are also made on a foundation of diet, exercise, and stress regulation as well as careful use of vestibular habituation exercises to lessen head motion sensitivity (13,87,93).

## THE WORK AHEAD

The Association for Migraine Disorders met in October 2020 to discuss gaps in knowledge about vestibular migraine. The 7 gaps are summarized as follows: 1) Lack of a universally accepted range of phenotypes, 2) Continued underdiagnosis, 3) Lack of objective markers, 4) Lack of understanding of the pathophysiologic mechanism, 5) Gender disparity in migraine-related phenotypes, 6) Lack of a methodical treatment plan, 7) Need for patient-reported outcome measures to assess the efficacy of treatment (94).

The evolution of migraine-related vestibular symptoms away from artery-centric terms such as “basilar migraine,” and “basilar-type migraine,” towards more general terms like “migraine with brainstem aura,” and now vestibular migraine, reflects the observation that vestibular migraine cannot be adequately explained by arterial pathology (6,40). The developing negative data on arterial pathology was not replaced with a testable new model, however, but ended up treating vestibular migraine as a primary disorder of no known structural cause (5). Currently, vestibular migraine is diagnosed based on symptoms rather than mechanisms. If a pathological mechanism were to be found, it could produce a variety of symptoms, creating an iterative cycle of defining what vestibular migraine really is. It could turn out to be like Meniere’s disease, which has multiple contributing vulnerabilities and instigators.

There is now a growing literature indicating that cerebral venous congestion syndromes from both intracranial and extracranial etiologies cause a variety of neurological symptoms including headaches, vertigo, disorientation, obtundation, visual loss, tinnitus, and autonomic dysregulation (95,96). Because the rarity of intracranial venous stenosis cannot explain the high prevalence of vestibular migraine, it may be time to look at cases of extracranial venous stenosis. Emerging case series indicate that extracranial venous compression can be associated with any of the symptoms that are associated with intracranial venous compression but happening in areas of high freedom of motion such as the neck, and that associated symptoms can be relieved by decompressing the obstruction (97–99). Digital subtraction venography has shown that compression of the internal jugular vein in patients with Meniere’s disease can be reversed with percutaneous transluminal venoplasty and relieve vertigo (100,101).

As we evolve our paradigms on vestibular migraine, progress may not be a straight line but more like an ascending spiral that allows us to see old ideas from new vantage points. In light of what modern tools have shown us about intravascular flow, could “humors,” outside the head cause intracranial pressure dysregulation due to vascular congestion as has been recently proposed for extracranial venous compression (102–104)? Could atmospheric pressure effects like “south winds,” affect the pressure in the inner ear, similar to how the hair cells in the lateral lines in fish detect pressure on their bodies (105)? Could it appear that migraine emanates from the ear not because of a shared arterial supply between the trigeminal nerve and the inner ear but because of a shared venous drainage (106,107)? While we stand on the shoulders of giants to see further, being mindful of where our big ideas came from can help us to look in the right direction.

## FUNDING SOURCES

The author received no financial support for the research, authorship, and/or publication of this article.

## CONFLICTS OF INTEREST

None declared.

## DATA AVAILABILITY STATEMENT

All data presented in this paper are accessible through the references. Data sharing is not applicable to this article as no datasets were generated or analyzed during the current study.
